# A Coordinated Data Analysis of Four Studies Exploring Age Differences in Social Interactions and Loneliness During a Global Pandemic

**DOI:** 10.1093/geronb/gbae086

**Published:** 2024-05-18

**Authors:** Shevaun D Neupert, Eileen K Graham, Destiny Ogle, Sumbleen Ali, Daisy V Zavala, Reilly Kincaid, MacKenzie L Hughes, Rita X Hu, Toni Antonucci, J Jill Suitor, Megan Gilligan, Kristine J Ajrouch, Stacey B Scott

**Affiliations:** Department of Psychology, North Carolina State University, Raleigh, North Carolina, USA; Department of Medical Social Sciences, Feinberg School of Medicine, Northwestern University, Chicago, Illinois, USA; Department of Sociology, Purdue University, West Lafayette, Indiana, USA; Department of Human Ecology, SUNY Oneonta, Oneonta, New York, USA; Department of Psychology, Stony Brook University, Stony Brook, New York, USA; Department of Sociology, Purdue University, West Lafayette, Indiana, USA; School of Psychology, Georgia Institute of Technology, Atlanta, Georgia, USA; School of Social Work, University of Michigan, Ann Arbor, Michigan, USA; Department of Psychology, University of Michigan, Ann Arbor, Michigan, USA; Department of Psychology, University of Michigan, Ann Arbor, Michigan, USA; Institute for Social Research, University of Michigan, Ann Arbor, Michigan, USA; Department of Sociology, Purdue University, West Lafayette, Indiana, USA; Department of Human Development and Family Science, University of Missouri, Columbia, Missouri, USA; Institute for Social Research, University of Michigan, Ann Arbor, Michigan, USA; Department of Sociology, Eastern Michigan University, Ypsilanti, Michigan, USA; Department of Psychology, Stony Brook University, Stony Brook, New York, USA

**Keywords:** COVID-19, Quantitative methods, Social interactions

## Abstract

**Objectives:**

Examining loneliness and social isolation during population-wide historical events may shed light on important theoretical questions about age differences, including whether these differences hold across different regions and the time course of the unfolding event. We used a systematic, preregistered approach of coordinated data analysis (CDA) of 4 studies (total *N* = 1,307; total observations = 18,492) that varied in design (intensive repeated-measures and cross-sectional), region, timing, and timescale during the first year of the coronavirus disease 2019 pandemic.

**Methods:**

We harmonized our data sets to a common period within 2020–2021 and created a common set of variables. We used a combination of ordinary least squares regression and multilevel modeling to address the extent to which there was within- and between-person variation in the associations between social isolation and loneliness, and whether these associations varied as a function of age.

**Results:**

Within- and between-person effects of social interactions were negatively associated with loneliness in 1 study; in follow-up sensitivity analyses, these patterns held across early and later pandemic periods. Across all data sets, there was no evidence of age differences in the within-person or between-person associations of social interactions and loneliness.

**Discussion:**

Applying the CDA methodological framework allowed us to detect common and divergent patterns of social interactions and loneliness across samples, ages, regions, periods, and study designs.

Developmental studies involve comparing individuals of different ages at a single time or comparing the scores from repeatedly sampling the same individuals across time, often with an aim of understanding patterns of change which may represent development, aging, adaptation, fluctuation, stability, growth, or decline (Baltes, 1968). The embedding of study design and data collection in the contexts of time and place, however, has received relatively less attention despite long-known influences of, for example, age, period, and cohort effects on findings (Atherton et al., 2023; Schaie, 1965). Indeed, it may be impossible to disentangle these influences in a single cohort or longitudinal study. However, in the field of aging research, the wealth of studies with common constructs over different timescales, fielded across different decades, regions, and cohorts, offers the potential to address these influences and uncover new theories of psychological and social aging (National Institute on Aging, 2021). This endeavor has significant challenges (e.g., harmonizing measures, siloed publications, and independent research teams). The current study addressed these challenges by applying the coordinated data analysis (CDA) approach and incorporated period effects (i.e., the coronavirus disease 2019 [COVID-19] pandemic as an external factor that is experienced by all groups in the population at a particular period in history; Yang & Land, 2006) to the question of age differences in the effects of social interactions on loneliness, leveraging rich data from four studies conducted during the first year of the COVID-19 pandemic in the United States. Figure 1 displays dates in 2020–2021 along the *X*-axis, with key historical dates marked across this period in the figure (see Supplementary Table 1). Overlaid on the right-most *Y*-axis are the national COVID-19 hospitalizations in red (The Atlantic, 2023). The left-most *Y*-axis depicts the dates of data collection for four studies (Within-Family Differences Study [WFDS]; Social Relations Study [SRS]; Einstein Aging Study [EAS]; Daily COVID-Fall [DCF]), with intensive repeated-measures data color coded in yellow bars and cross-sectional data color-coded in purple bars.

**Figure 1.**
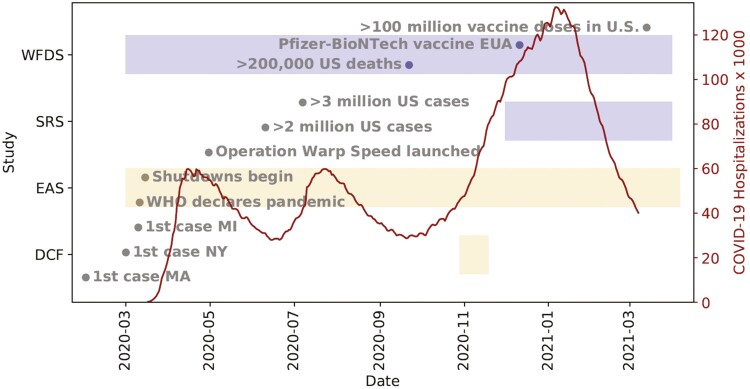
Timeline of Data Collection for Each Study Mapped onto Major COVID-19 Events.

Tremendous advances have been made through large, nationally representative panel studies (e.g., Health and Retirement Study, Midlife in the United States, English Longitudinal Study of Ageing [ELSA], German Socio-Economic Panel), and yet much of the foundational knowledge about aging has been developed in small- to mid-sized, geographically circumscribed samples with rich behavioral, biological, and psychological cross-sectional or longitudinal data (e.g., Harvard Study of Adult Development, Normative Aging Study, Hawaii Longitudinal Study of Personality and Health, Dunedin Study, SRS, WFDS). The National Institute on Aging (NIA) has described these kinds of studies as “deeply phenotyped” and has invested significant resources in their development and maintenance (National Institute on Aging, 2021). However, most deeply phenotyped studies are published in isolation, in single-study papers, and cross-study comparisons typically occur in literature reviews, which cannot test the systematic effects of many theory-relevant features (e.g., cohort, geography, time period). Although valuable, conducting a traditional meta-analysis will not resolve this issue because of study-specific differences in design and analytic decisions. To leverage these resources and advance knowledge of development, cross-study comparisons of a common research question need to be made directly. Unlike larger, nationally representative, cohort studies, data sets for most deeply phenotyped studies are not publicly available, but are locally housed at the study PIs’ institutions. CDA is a promising avenue to maximize collaboration of these valuable data sets that cannot be shared and have varying designs (e.g., ecological momentary assessments [EMAs], daily diaries, cross-sectional). CDAs do not require direct data sharing, and are often promoted as ways to harmonize and analyze data sets that would otherwise remain siloed. Intentionally focusing on these smaller, locally controlled studies allows their contributions to be featured and included in multistudy replication efforts. Thus, by applying the CDA framework to unshareable (i.e., locally controlled, not publicly available) deeply phenotyped studies of aging, we are building an opportunity for these studies to be included in the open science/replicability movement.

## Coordinated Data Analysis

CDA is one approach for direct, cross-study comparisons within a larger family of methodologies known as integrative data analysis (Hofer & Piccinin, 2009; Curran & Hussong, 2009; Graham et al., 2022). In CDA, the project team first identifies a key foundational question to answer along with candidate studies that meet predetermined minimum inclusion criteria for analysis. The key variables are then conceptually harmonized at the construct level, and uniform models are estimated alongside one another at the individual study level, allowing each data set to account for its specific nuances and idiosyncrasies (e.g., different timescales). Results are then synthesized, often (but not always) using the tools of meta-analysis to draw conclusions about both the target hypotheses and the extent to which findings were replicated across studies (Graham et al., 2022; Mroczek et al., 2022; Weston et al., 2020; Willroth et al., 2022). These approaches have gained popularity within the last decade as one way of addressing issues of replicability within subfields of the psychological sciences that rely on long-term longitudinal data (Beck et al., 2023; Graham et al., 2017; Graham et al., 2020; Graham et al., 2022; Luo et al., 2022; Turiano et al., 2020; Weston et al., 2020; Willroth et al., 2022; Wood et al., 2018; Yoneda et al., 2021). Key strengths of this approach are that study teams are not required to share data and can run their analyses in-house. This means that the leading experts of those data sets are responsible for the cleaning, transformation, and analysis of their own data, which can benefit a project both by introducing fewer opportunities for human error and by reducing inclusion barriers because data sharing is not required (Graham et al., 2022; Weston et al., 2020). Because CDA does not require the same study designs (i.e., frequency, duration) or analytic approach across studies, it can be an optimal way to directly compare studies that capture developmental processes under the developmentally relevant but atypical study design and data collection conditions of a natural experiment (VandenBos, 2015).

## Embedding Development in Context

Examining responses to population-wide historical events may shed light on important theoretical questions about age differences in social and emotional experiences. Bronfenbrenner’s ecological systems theory (1979) posits that an individual’s environment is characterized by five concentric systems of proximal and distal bidirectional influences. The chronosystem entails patterning environmental events over the life course, including historical events that may function as period effects. Period effects can impact development when environmental and historical events that occur around data collection modulate participant responses or measures in ways that diverge from normative patterns of theoretically expected age differences (Yang & Land, 2013).

Although the COVID-19 pandemic has been described as a unique, once-in-a-generation experience (Office of the Governor of Illinois, 2023, January 31), from a life-span developmental perspective, it fits as an example of the macro-level events that are part of modern life, including major economic recessions and extreme weather events, and affected the daily life of individuals across the life span who were living at that time in history (Martire & Isaacowitz, 2021). Using longitudinal data from the National Study of Daily Experiences over 10 years, Almeida et al. (2020) showed that daily life is more stressful in midlife for later-born cohorts, reflecting the potential impact of the Great Recession of 2008. Segerstrom et al. (2023) used two longitudinal data sets from two different U.S. states and showed that pandemic severity associated with time and place was critical in understanding changes in cognitive difficulties, depressive symptoms, and stress. Identifying period effects by examining developmental data from various places and points in time is necessary to draw conclusions regarding age-related differences in pandemic effects on psychological well-being. Understanding how these period effects may vary depending on time and place requires multiple samples across time and regions. The current study addresses period effects by using time as a harmonizing feature and integrating auxiliary community and regional data to uncover common and divergent patterns observed across the samples, ages, regions, and phases of the first year of the pandemic.

## Why Study Loneliness in Aging?

Loneliness is a subjective state that reflects people’s experiences and evaluations of a social situation. Feelings of loneliness arise due to discrepancy between one’s desired versus actual quality of social interactions (Hawkley et al., 2008). Under typical conditions before the pandemic, social interactions were negatively associated with loneliness (Kobayashi & Steptoe, 2018). Graham et al. (2024) found a nonlinear (U-shaped) trajectory of loneliness across the adult life span using nine independent data sources collected prior to the onset of COVID. They found elevated loneliness levels among older adults and young adults, and that social interactions were among the most robust predictors of loneliness which did not differ by age (was related to overall loneliness intercepts, but not slopes). The current study builds upon this work by exploring whether the findings from typical conditions would hold under the unfolding, unpredictable, and volatile conditions of the first year of the pandemic, during which the health risk and local norms differed across time and U.S. region.

Following the COVID-19 pandemic, the Surgeon General’s 2023 report declared an epidemic of loneliness and isolation, and that older adults reported the highest rates of social isolation (Office of the U.S. Surgeon General, 2023). Using the ELSA data set, DiGessa and Zaninotto (2023) compared older adults who reported being socially isolated before the COVID-19 pandemic with those who reported being socially engaged before the pandemic. The findings revealed that both groups reported decreased life satisfaction, quality of life, and higher levels of loneliness, depression, and anxiety during the pandemic. However, socially engaged older adults reported significantly greater deteriorations in these outcomes. The COVID-19 pandemic and accompanying public health control measures substantially increased the prevalence of loneliness, especially among adults aged 65 and over, according to a recent meta-analysis of over 30 studies covering 28,050 participants (Su et al., 2023). However, levels of loneliness were not constant across the pandemic, which suggests the potential importance of considering multiple period effects. Prevalence estimates of loneliness among older adults were significantly higher for those studies conducted later in the pandemic (more than three months after the pandemic started) as compared to studies conducted within the first 3 months of the pandemic (Su et al., 2023).

When considering age differences in loneliness, it is important to keep in mind that younger adults typically have much larger social networks than older adults (Wrzus et al., 2013) and thus had more to lose when lockdowns started. Older adults tend to have smaller and more carefully pruned social networks than younger adults do (Alwin et al., 2018). One possible reason for this could be that younger adults’ networks often include many social partners who were not chosen freely, but rather were incorporated into the network because of relationships from work or an offspring’s school network (Alwin et al., 2018). In contrast, older adults’ social networks tend to be based on relatively larger percentages of emotionally close social partners than younger adults’ networks (Alwin et al., 2018). When COVID-19 restrictions began in the United States in March 2020, the impact of the lockdowns on the experience of loneliness among older adults may have been considerably lower compared to younger adults. Indeed, several studies have shown that older adults’ mental well-being fared much better than expected; older adults reported less emotional distress than younger adults during the first months of the pandemic (Carstensen et al., 2020; Klaiber et al., 2021).

## Present Study

We used the CDA methodological framework (Hofer & Piccinin, 2009; Graham et al., 2022), which allowed us to explore common and divergent patterns observed across the samples, ages, regions, and periods. Our preregistered hypotheses (https://osf.io/n9afu/registrations) targeted between-person differences as well as within-person processes. H1a: For all studies, we expected that people who reported less social interaction would report higher loneliness compared to people who reported more social interaction. H1b: For the intensive repeated-measures studies, we expected that on occasions when people reported lower social interaction they would also report higher loneliness. H2: In addition, we expected an interaction between age and social interaction, where the between-person (H2a) and within-person (H2b) relationship between social interaction and loneliness would be stronger for younger relative to older adults. H3: We further expected that this interaction effect would be more pronounced in studies with larger age ranges. H4: In studies that took place during high hospitalization periods, we expected to observe a stronger negative association between social interactions and loneliness.

## Method

All research questions, hypotheses, and planned analyses were preregistered on the Open Science Framework (OSF; https://osf.io/n9afu/registrations). Analyses for the current study were completed using four independent existing data sets. Individual study analyses were conducted using R version 4.3.1 (R Core Team, 2013), Stata (StataCorp, 2017), and SAS 9.4 (SAS 9.4). Code for all analyses is available at https://osf.io/n9afu/.

### Studies

The four studies used for the current study were identified from investigators who participated in a breakout session during the NIA workshop called “Deeply Phenotyped Longitudinal Studies of Aging: Opportunities for Coordination and Collaboration” which was held on February 25–26, 2021 (National Institute on Aging, 2021). At the end of the breakout session, investigators who wanted to join this project formed a working group and invited early career researchers from their respective teams to join. For inclusion in the current analyses, data collection needed to be completed during the first year of the COVID-19 pandemic and the study needed to assess social interactions and loneliness within the context of the pandemic. Data collection dates ranged from March 2020 through March 2021. Study-specific demographics are displayed in Table 1. See Figure 1 for the timeline of data collection.

**Table 1.** Sample Characteristics From Four Studies

Variable	DCF (*N *= 218)		EAS (*N* = 147)		SRS-COVID (*N* = 141)		WFDS (*N* = 801)	
	Range	*M* (*SD*)/%	Range	*M* (*SD*)/%	Range	*M* (*SD*)/%	Range	*M* (*SD*)/%
Age	21–78	49.08 (14.84)	72–96	78.74 (5.04)	64–94	73.50 (7.38)	18–80	44.21 (17.50)
Gender—female		58.33%		71.28%		63.38%		58.93%
Race—White		74.77%		47.18%		57.04%		82.53%
Marital status—married/partnered		50.92%		31.44%		49.30%		51.69%
Education	5–25	14.51 (3.10)	2–25	15.22 (3.48)	5–17	14.35 (2.42)	1–7	5.51 (1.34)
Income	1–6	3.33 (1.13)	1–4	2.50 (0.68)	1–7	4.60 (1.41)	0.083–0.50	3.46 (1.91)
	$833 or less	6.88%	less than $1,250	9.23%	< $500	0.70%	Less than $2,500	26.96%
	$833 to $2,083	14.22%	$1,251-$2,500	32.82%	$501–1,000	7.04%	$2,500–$4,167	9.36%
	$2,083 to $4,167	33.94%	Greater than $2,500	56.41%	$1,001–2,000	16.20%	$4,168–$6,250	13.11%
	$4,167 to $8,333	30.28%	Refused	1.54%	$2001–3,000	23.24%	$6,251–$8,333	12.73%
	$8,333 to $20,833	13.30%			$3,001–5,000	19.01%	$8,334–$12,500	16.60%
	$20,833 or more	1.38%			$5,001–10,000	28.17%	More than $12,501	21.11%
					>$10,000	5.63%		
Employment—employed		70.98%		5.64%		14.08%		68.29%
Self-rated health (Raw)	1–5	3.61 (0.97)	2.93–5.00	4.57 (0.51)	1–5	3.25 (1.14)	1–5	3.68 (0.93)
Self-rated health (Standardized)	−2.63 to 1.38	0 (0.98)	−3.18 to 0.83	-0.003 (0.99)	−1.98 to 1.54	0.00 (1.00)	−2.87 to 1.37	−0.032 (0.98)
Loneliness (Raw)	1–4	1.57 (0.75)	0–99.17	17.27 (19.92)	1–4	1.73 (0.97)	1–4	1.55 (0.87)
Loneliness (POMP score)	0–10	1.91 (2.51)	0–9.92	1.72 (1.99)	0–10	2.42 (3.23)	0–10	1.86(2.89)
Social interactions (Raw)	0–50	3.61 (4.63)	0–1.00	0.75 (0.24)	1–4	2.09 (1.02)	1–7	4.24 (2.21)
Social interactions (POMP score)	0–6.44	0.72 (0.93)	—	—	0–10	3.64 (3.41)	0–10	5.39 (3.68)

*Notes*: DCF = Daily COVID-Fall; EAS = Einstein Aging Study; POMP = percent-of-maximum-possible; SRS-COVID = Social Relations Study—COVID; WFDS = Within-Family Differences Study.

#### Daily COVID-19 Fall

Daily COVID-19 Fall (DCF) is a microlongitudinal 21-day daily diary study (Pearman et al., 2022). Participants identified as either White or Black/African American, and were recruited through Amazon Mechanical Turk and Qualtrics. Participants were from 36 U.S. states. Daily diaries began on October 29, 2020, and ended on November 18, 2020.

#### Einstein Aging Study

The EAS is an ongoing longitudinal study which includes 14 days of up to four EMAs each day (Pasquini et al., 2022; Zhaoyang et al., 2022). Participants were recruited from Bronx County, NY, registered voter lists. EMA data for the present analyses were drawn from participants who completed momentary assessments during the first year of the pandemic. The analytic sample included data collected between March 2020 and March 2021.

#### Social Relations Study—COVID

The SRS-COVID was a COVID-19 supplement of the Detroit Area Wellness Network project. Participants were recruited from an existing longitudinal cohort study, the SRS, which began in 1992 (Antonucci & Akiyama, 1994). Participants were recruited from Southeast Michigan using an address-based and respondent-driven sampling strategy. Cross-sectional data for the present analyses were collected between December 2020 and March 2021.

#### Within-Family Differences Study

The WFDS is an ongoing longitudinal study of intergenerational relationships which recruited a probability sample of women ages 65–75 with two or more children in the greater Boston area (Suitor et al., 2023). Cross-sectional data for the present analyses were drawn from the WFDS-III, conducted from March 2020 to March 2021.

##### Measures

Supplementary Table 2 provides the items used in each study. Later, we describe the harmonization for each of the variables used in the analysis.

###### Loneliness

Loneliness was assessed at different timescales in each study (e.g., EAS participants rated their momentary loneliness up to 4 times per day). However, for each study, participants were given a single item that captured the frequency of experienced loneliness (e.g., how often have you felt lonely), with response options ranging from (1 = rarely to 4 = all of the time) to (1 = <1 day to 4 = 5–7 days). For comparability and ease of interpretation across data sets, all loneliness scores were transformed into percent-of-maximum-possible (POMP) scores such that all studies’ results below report loneliness on the same 0–10 scale.

###### Social isolation

Each study used different items to collect how much social contact participants had during the study period. EAS used a binary response option, with participants simply rating whether (or not) they had any social interactions since the last survey. SRS and WFDS asked participants to rate the frequency with which they had social interactions (e.g., every day, several times a week, etc.). Lastly, DCF provided an open-ended prompt asking participants to enter the number of people (besides the individuals they live with) they interacted with in the past 24 hr. With the exception of the binary responses collected in EAS, these responses were POMP scored for cross-study comparability. DCF’s maximum response was constrained to +2*SD*s.

###### Age

Age was calculated in each study from year of birth at a given wave, and then centered at the study-level grand mean.

##### Covariates

*Gender* (1 = female, 0 = male), *race* (1 = White, 0 = non-White), *marital status* (1 = married/partnered, 0 = all others), and *employment status* (1 = employed, 0 = not employed) were harmonized across studies by transforming to binary categories. *Education*, *income*, and *self-rated health* were standardized around each study’s mean.

##### Individual study analysis

Due to the heterogeneity in study designs, our preregistered analytic plan (https://osf.io/n9afu/registrations) included different modeling approaches for the cross-sectional and intensive repeated-measures data sets. All models were adjusted for sex, race, marital status, education, income, employment status, and self-rated health. The fully adjusted models are reported later. Results from the unadjusted models can be found in Supplementary Table 3. We applied multilevel models (MLMs) to the intensive repeated-measures data sets (i.e., DCF, EAS) with nested data. The two-level equation used in DCF and the three-level equation EAS momentary data can be found in Supplementary Material.

The MLMs were estimated with random slopes. In the event that models failed to converge with the random slopes, our preregistration outlined that these parameters would be removed. In our intensive repeated-measures studies, this only occurred for DCF. Thus, for the current paper, we report models for DCF without random slopes.

### Meta-analysis

As this was a relatively small CDA (*k* = 4) with diverging analytic approaches, it was not appropriate for us to conduct meta-analyses for this project. Instead, upon completion of the individual study analyses, all results were compiled into a table (see Table 2) and are reported later.

**Table 2.** Results From Four Studies—Loneliness Predicted by Social Interactions, Age, and Covariates

Variables	Multilevel models						OLS Regression Models					
	DCF			EAS			SRS			WFDS		
	Est	*SE*	*p*	Est	*SE*	*p*	Est	*SE*	*p*	Est	*SE*	*p*
*Fixed effects*												
Intercept	2.23***	0.59	<.001	0.65	.68	.335	2.44**	0.76	.002	2.668***	0.386	<.001
BP social interactions	−0.02	0.21	.93	−1.89**	.64	.004	−0.06	0.08	.44	−0.0258	0.027	.344
WP social interactions	−0.04	0.03	.14	−0.14***	.04	<.001	—	—		—	—	
Age	-0.04**	0.02	.01	-0.004	.03	.895	0.001	0.06	.99	−0.0161	0.011	.133
Age × BP social interactions	0.00	0.01	.98	0.01	.11	.919	0.003	0.01	.79	0.00049	0.002	.77
Age × WP social interactions	0.00	0.00	.19	0.01	.01	.091	—	—		—	—	
Gender	−0.53	0.38	.17	0.77	.34	.026	0.73	0.62	.24	0.162	0.205	.429
Race	0.55	0.45	.22	0.14	.30	.650	−0.04	0.61	.95	0.0655	0.290	.822
Marital status	−0.40	0.38	.29	0.08	.36	.818	−0.08	0.69	.91	−1.124***	0.274	<.001
Education	0.18	0.17	.29	−0.06	.16	.701	−0.03	0.31	.92	−0.0309	0.123	.801
Income	−0.08	0.20	.70	0.10	.17	.570	−0.56	0.36	.12	−0.125	0.162	.441
Employment	−0.35	0.49	.48	0.77	.64	.230	−1.25	0.81	.12	−0.418	0.230	.07
Self-rated health	−0.28	0.20	.17	*−^a*	—	—	−0.32	0.28	.26	−0.368**	0.120	.002
*Random effects*												
Intercept (person)	5.72***	0.63	<.0001	3.78	.40	<.0001	—	—	—	—	—	—
Intercept (day)	—	—	—	0.36	.02	<.0001	—	—	—	—	—	—
Cov (Int, WP social interaction slope)	−*^b*	—	—	−0.15	.07	.0395	—	—	—	—	—	—
WP social interaction slope	−*^b*	—	—	0.12	.03	<.0001	—	—	—	—	—	—
Residual	2.46***	0.07	<.0001	0.93	.01	<.0001	—	—	—	—	—	—
*N* (Persons)	191			194			141			801		
*N* (Observations)	2,879			14,671			—			—		

*Notes*: BP = between person; DCF = Daily COVID-Fall; EAS = Einstein Aging Study; POMP = percent-of-maximum-possible; SRS-COVID = Social Relations Study-COVID; WFDS = Within-Family Differences Study; WP = within person. Social interactions were POMP scored. Age was centered at each study’s mean. Reference groups were as follows: Gender (1 = Female, 0 = Male) was assessed in DCF and EAS by asking participants to state the gender they currently identify with. Although the response format allowed for the inclusion of nonbinary conforming individuals, the data distribution was approximately binary. As such, individuals in these studies identifying female (cis or trans) and male (cis or trans) were coded accordingly. For SRS, participants were asked to indicate whether they were male or female, and for WFDS, participants’ mothers were asked to confirm the participants’ gender at the previous wave. Marital status (1 = Married/partnered, 0 = All others). Race (1 = White, 0 = Non-White), Employment (1 = Employed, 0 = Unemployed). Education, income, and self-rated health were *z*-scored. Results for DCF and EAS are drawn from multilevel models of intensive longitudinal data, thus these include separate predictors for the between-person and within-person effects of social interactions. Within-person effects were person mean centered; between-person effects were grand mean centered. The footnote *^a* refers to EAS only *N* = 129 had available data for self-rated health, which reduced our statistical power to reliably detect an effect. Here, we reported the EAS model unadjusted for self-rated health (*N* = 194). A summary of EAS model with self-rated health included can be found in Supplementary Table 4. These models include random effects, except where indicated with a *^b*. The model for DCF that included a random slope did not converge, thus the reported model includes only a random intercept. Results for SRS and WFDS are drawn from ordinary least squares regression of cross-sectional surveys, thus these include the between-person effect of social interactions, and no random effects.

## Results

The results of H1a, H1b, H2a, and H2b are presented in Table 2 and described as follows.

*Between-person associations between social interaction and loneliness* (H1a).

In EAS, there was a significant between-person effect where people with fewer social interactions reported higher levels of loneliness. In the three other studies, we did not find evidence that individual differences in social interactions predicted individuals’ levels of loneliness.

*Within-person associations between social interaction and loneliness* (H1b: DCF and EAS only).

Unconditional models indicated that approximately 41% of the variance in social interactions and 69% of the variance in loneliness was between persons in DCF. Unconditional model results indicated that approximately 30% of the variance in momentary social interactions and 75% of the variance in momentary loneliness was between persons in EAS. In EAS, there was a significant within-person effect, suggesting that at times with more social interactions than usual, participants reported less loneliness. The within-person effect of social interaction on loneliness was not significant in DCF.

*Between-person interaction of age and social interaction* (H2a)

For all four studies, the interaction between age and between-person social interaction was not significant.

*Cross-level interaction of age and within-person social interaction (H2b: DCF and EAS Only)*.

There was no evidence of a cross-level interaction in either study.

*Studies with larger age ranges would have more pronounced interaction effects* (H3).

We did not test this hypothesis because there was no evidence of an interaction in any study. It is worth noting that the studies with the wider age ranges (DCF and WFDS included both younger and older adults) actually had the smallest interaction estimates.

*For studies that took place during high hospitalization periods, we expected to observe a stronger negative association between social interactions and loneliness* (H4).

Given that a significant association between social interactions and loneliness at the between- or within-person levels was only observed in EAS, we could not test this prediction across studies. However, because EAS collected data throughout the period between March 2020 and March 2021 in which individual participants participated for approximately 2 weeks, we conducted an unregistered follow-up analysis (see OSF project page: https://osf.io/n9afu/) using the reporting template from Willroth and Atherton (2024) that tested whether different patterns emerged depending on when individuals were observed during the pandemic. The results of this follow-up analysis are presented in Table 3. We operationalized “early pandemic” constituting the first wave of COVID-19 hospitalizations in the New York City area (New York City Department of Health and Mental Hygiene, 2023). We operationalized “later pandemic” based on changes in local restrictions (i.e., New York City enters Phase One of Reopening on June 8, 2020). In both the early and later pandemic periods (Table 3), the pattern was consistent with the overall results presented in Table 2; individuals with more social interactions reported less average levels of loneliness compared to individuals with fewer social interactions, and at moments with more social interactions than usual, participants reported less loneliness compared to moments with fewer social interactions than usual. Consistent with the overall study results, age interactions were not observed in either period.

**Table 3.** Unregistered Follow-up EAS Analysis Comparing Time in Pandemic

	March 2020–June 2020			July 2020—March 2021		
Fixed effects	Est	*SE*	*p*	Est	*SE*	*p*
Intercept	0.80	1.06	.451	0.64	0.73	.3797
Social interactions: BP	−1.96	1.04	.0626	−1.30	0.68	.0596
Social interactions: WP	−0.13	0.08	.1161	−0.13	0.03	.0002
Age	0.02	0.04	.7143	−0.02	0.03	.6051
Age × Social interaction BP	−0.06	0.14	.6651	0.25	0.15	.0868
Age × Social interaction WP	0.03	0.02	.0778	0.01	0.01	.3509
Gender	0.99	0.54	.0724	0.47	0.37	.213
Race	−0.36	0.46	.431	0.55	0.33	.0964
Marital status	−0.40	0.57	.4822	0.40	0.39	.3059
Education	−0.33	0.26	.2165	0.06	0.17	.7287
Income	−0.01	0.24	.9755	0.18	0.19	.3655
Employment	1.06	0.95	.2667	0.35	0.69	.6152
*Random effects*						
Intercept (person)	3.63	0.62	<.0001	3.52	0.43	<.0001
Intercept (day)	0.29	0.02	<.0001	0.40	0.02	<.0001
Cov (Int, WP social interaction slope)	−0.17	0.16	.2751	−0.17	0.06	.0098
WP social interaction slope	0.30	0.07	<.0001	0.04	0.02	.0064
Residual	0.85	0.02	<.0001	0.80	0.01	<.0001
Persons	80			147		
Observations	4,778			9,893		

*Notes*: BP = between person; EAS = Einstein Aging Study; WP = within person. Models reported here use the same specifications and predictors as those reported in Table 2 for EAS.

## Discussion

The goal of the current study was to apply the CDA methodological framework (Graham et al., 2022; Hofer & Piccinin, 2009) to detect common and divergent patterns of social interactions and loneliness observed across samples, ages, regions, and periods. Although our preregistered hypotheses were only partially supported, we did find common themes across the studies. There was a consistent lack of evidence at both the between-person and within-person levels for an interaction between social interaction and age predicting loneliness. In line with our between-person predictions, in one study, we found that people who reported more social interactions reported lower levels of loneliness compared to people with less social interactions. Our within-person prediction was supported in one study; occasions of increased social interaction were associated with decreased loneliness.

One of the unique contributions and challenges of this endeavor was to use time (i.e., date of data collection) as a harmonizing feature for which to test whether predictions based on nonpandemic conditions held. The undulating waves of hospitalizations over the first year and unfolding regional and national events highlighted in Figure 1 speak to the varying health risks and social constraints during 2020–2021. One intensive repeated-measures data set (EAS) and one cross-sectional (WFDS) data set fielded data continuously. DCF collected data at the beginning of the third wave, and SRS collected data throughout the third wave. Given the consistency of findings across three data sets (WFDS, DCF, and SRS), our results are not due to systematic differences associated with a single wave of pandemic severity or the local conditions of a single study. In EAS, however, our predictions for between- and within-person effects were supported.

Hoppmann and Pauly’s (2022) life-span psychological perspective on solitude offers at least two relevant explanations. First, here, we operationalized our predictor as presence and frequency of social interactions; however, solitude (i.e., absence of social interaction; often coded the opposite way as our EAS momentary social interaction variable) is also relevant. Solitude has been associated with increased loneliness (Hoppmann et al., 2021) but also greater positive experiences (increased calm, near absence of negative affect; Lay et al., 2019). It is possible that the differing patterns we found for EAS relative to the other studies may reflect the varying effects found for solitude. Second, Hoppmann and Pauly raise the methodological challenge of understanding the effects of social interactions (or the absence of social interactions, as in solitude), which may operate differently over micro- and macro-timescales. In EAS, we observed effects when querying older adults about social interactions over the last few hours and current loneliness (micro-time), but in the age-heterogeneous samples reporting social interactions and loneliness today and more globally, we did not observe these effects.

Across all data sets, we found no evidence of age differences in the within-person or between-person relationships between social interactions and loneliness. These results are in line with Graham et al. (2024), who found evidence for a U-shaped trajectory of loneliness across the adult life span: social isolation was only associated with loneliness intercepts (overall levels), but not slopes suggesting the isolation is more closely associated with persistent loneliness but not with age-graded change in loneliness. With respect to the current study findings, it is possible that the period effect of the first year of the pandemic may flatten or blunt some associations, as suggested by Strength and Vulnerability Integration (SAVI; Charles, 2010; Charles & Piazza, 2023). Specifically, age differences in emotional well-being are predicted to vary based on proximity to the stressor, with age differences occurring before and after, but not during, the stressor (Charles & Piazza, 2023). During or very near the onset of an event, older and younger adults may be similar (i.e., age differences are not observed). However, as time passes, age differences that may have existed before the event return (Charles, 2010; Scott et al., 2017). In follow-up analyses in EAS, we compared the “early” period when the highest peak of hospitalizations occurred in the data collection region (Bronx, NY) between March and June 2020 to the “later” period. We found no evidence that age effects differed between these periods, suggesting that the blunting effect was durable across the first year of the pandemic. Further, the between- and within-person effects of social interactions on loneliness held across both periods. These findings underscore that our understanding of developmental processes, including age differences, that were developed outside of major historical events may not hold (Scheidel, 2017). Prior work supporting SAVI’s predictions regarding proximity of events and age differences was based on daily stressor events, which likely have a very different time course of the event itself and response than the pandemic.

From the current study, we can also draw several methodological conclusions. Although methods of integrative data analysis are powerful tools for synthesizing data across independent data sets, the true CDA approach, as defined by Graham et al. (2022), can be difficult to apply to deeply phenotyped longitudinal studies. We discovered this to be particularly true in the current study because the inclusion criteria required data to be collected during a very constrained time period (i.e., during the first year of the COVID-19 pandemic). Although each of the four studies we used was technically able to answer the key research questions of the current study, the design features of each study rendered it impossible for identical models to be estimated across the data sets. Thus, the investigative team reached the consensus to test the best possible model each data set could estimate given its specific design (e.g., OLS regression for cross-sectional data sets and MLMs for the intensive repeated-measures data sets). This allowed the intensive repeated-measures studies to maximize their available data. Future work using similarly unique data sets (e.g., diary data, EMA studies) should continue to explore ways to optimize model comparability, to improve methods for applying the tools of integrative data analysis, and provide a toolbox of methods for researchers to conduct robust, reproducible, and replicable analyses on these specialized data sets.

### Limitations and Future Directions

Although the CDA framework provides a robust test of underlying hypotheses and direct, cross-study comparisons, some limitations should be acknowledged. The current results were restricted to studies that collected data during the early COVID-19 time frame and had constructs that could reasonably be harmonized with one another. Although all four studies are longitudinal in nature, SRS and WFDS functioned as cross-sectional data sets in the current study because they had one assessment per person during the first year of the pandemic. We were additionally limited by the number of data sets and therefore could not conduct a reliable meta-analysis. Although the current results could not be directly compared via meta-analytic synthesis, our CDA approach provides unique puzzle pieces that, put together, paint a picture that drills down deeper than many CDAs that use large longitudinal panel studies are able to. In addition, the distinct data types (cross-sectional, daily diary, EMA) within the existing data sets allowed for direct comparisons of between-person effects in all studies. However, our ability to make within-person comparisons across longitudinal studies was limited.

We were not able to examine how the patterns we observed differed from prepandemic levels because this information was not available for all of our studies. However, a prior paper (Pasquini et al., 2022) focused on a subset of EAS participants who provided data during the very early pandemic period (February 2020—June 2020) and in a prior burst (primarily from 2019). There was evidence of significant and robust increases in average momentary loneliness from 2019 to the 2020 early pandemic period. Our current approach that focused on the first year of the pandemic allowed us to capture snapshots of within-person processes during this historical time period, but we are unable to make any assertions regarding potential long-term changes in trajectories of loneliness. Although we know that microlongitudinal fluctuations can be an important feature of macrolongitudinal changes (Scott et al., in press), future work that incorporates macrolongitudinal designs will be best suited to assess changes in the trajectory of loneliness from before to after the pandemic and the role of social interactions. Further, we do not expect these patterns to generalize outside of the United States, given our intentional context-specific focus that overlaid COVID-19 milestones and regional aspects to these period effects. In addition, it could be fruitful to test age interactions within a period of the life span. Although we did not find evidence of age interactions within the EAS data set, future studies could examine whether the patterns observed for social interaction and loneliness are consistent or varying within samples of older adults. Efforts to increase coordination and collaboration are essential for replication and testing the boundary conditions under which established patterns do and do not hold. Further, triangulating multicohort projects can open new lines of inquiry and discovery that would be impossible with a single study.

## Conclusion

Using the CDA methodological framework (Graham et al., 2022; Hofer & Piccinin, 2009), we explored common and divergent patterns across samples, ages, regions, and periods in social interactions and loneliness. Although the process of a CDA typically takes longer than a single-study investigation, there are valuable benefits regarding replication, yielding confidence in the findings. Across all intensive repeated-measures and cross-sectional data, there was no evidence of age differences in the within-person or between-person associations of social interactions and loneliness. By layering auxiliary contextual information such as hospitalizations over the period of interest, we can integrate chronosystem influences on development.

## Supplementary Material

gbae086_suppl_Supplementary_Material

## Data Availability

This study was preregistered. Access to the data and study materials are described in the preregistration (https://osf.io/n9afu/registrations). Analytic code can be found at https://osf.io/n9afu/.
