# Supplementary material for: A Coordinated Data Analysis of Four Studies Exploring Age Differences in Social Interactions and Loneliness During a Global Pandemic
Source: J Gerontol B Psychol Sci Soc Sci. 2024 May 18;79(8):gbae086. doi: 10.1093/geronb/gbae086 (PMC11247406; doi:10.1093/geronb/gbae086)
Supplement: gbae086_suppl_Supplementary_Material [file gbae086_suppl_supplementary_material.docx]

***The Journals of Gerontology Series B: Psychological Sciences and Social Sciences* Supplementary Material: Neupert et al. A Coordinated Data Analysis of Four Studies Exploring Age Differences in Social Interactions and Loneliness During a Global Pandemic.**

**Section 1: Supplemental Tables**

**Supplemental Table 1: Notable COVID-19 Events & Dates**

| **Event** | **Date** |
| --- | --- |
| First laboratory-confirmed COVID-19 case in the U.S. | 01-20-2020 |
| WHO determines outbreak constitutes a Public Health Emergency of International Concern (PHEIC) | 01-30-2020 |
| First COVID-19 case in Massachusetts* | 02-01-2020 |
| First COVID-19 death in the U.S. (but the link is not confirmed until April 21, 2020) | 02-06-2020 |
| First COVID-19 case in New York* | 03-01-2020 |
| First COVID-19 case in Michigan* | 03-10-2020 |
| The WHO declares COVID-19 a pandemic* | 03-11-2020 |
| The Trump Administration declares a nationwide emergency | 03-13-2020 |
| States begin to implement shutdowns to prevent the spread of COVID-19* | 03-15-2020 |
| The Trump Administration signs the Coronavirus Aid, Relief, and Economic Security (CARES) Act into law | 03-27-2020 |
| The White House extends all social distancing measures through the end of April 2020* | 03-28-2020 |
| The CDC announces new mask wearing guidelines and recommends that all people wear a mask when outside of the home | 04-03-2020 |
| Doctors and civil rights groups urge the CDC and the U.S. government to release race and ethnicity data on COVID-19 case-numbers to understand the impact of the virus on communities of color | 04-06-2020 |
| The U.S. is the country with the most reported COVID-19 cases and deaths | 04-10-2020 |
| Most states in the U.S. report widespread cases of COVID-19 | 04-12-2020 |
| The Trump Administration launches Operation Warp Speed* | 04-30-2020 |
| The unemployment rate in the U.S. is 14.7%, the highest since the Great Depression | 05-09-2020 |
| The recorded death toll from COVID-19 in the U.S. surpasses 100,000 | 05-28-2020 |
| The number of confirmed COVID-19 cases in the U.S. surpasses 2 million* | 06-10-2020 |
| The number of confirmed COVID-19 cases in the U.S. surpasses 3 million* | 07-07-2020 |
| HHS announces a plan to make COVID-19 vaccines free in the U.S. | 09-16-2020 |
| The reported death toll in the U.S from COVID-19 surpasses 200,000* | 09-22-2020 |
| FDA issues an EUA for the Pfizer-BioNTech COVID-19 vaccine* | 12-11-2020 |
| The recorded death toll from COVID-19 in the U.S surpasses 300,000 | 12-14-2020 |
| FDA issues an EUA for the Moderna COVID-19 vaccine | 12-18-2020 |
| More than 1 million COVID-19 vaccine doses have been administered to health care workers and older adults in long-term care facilities in the U.S. in 10 days | 12-24-2020 |
| The first case of the Alpha COVID-19 variant is detected in the U.S. | 12-29-2020 |
| One year anniversary of the first reported case of COVID-19 to the WHO | 12-31-2020 |
| The reported death toll from COVID-19 in the U.S. surpasses 400,000 | 01-18-2021 |
| CDC requires face masks to be worn by all travelers while on public transportation and inside transportation hubs | 01-20-2021 |
| The recorded COVID-19 death toll in the U.S. surpasses 500,000 | 02-21-2021 |
| FDA approves an emergency use authorization (EUA) for Johnson & Johnson’s one-shot COVID-19 vaccine | 02-27-2021 |
| CDC recommends people who are fully vaccinated against COVID-19 can safely gather with other fully vaccinated people indoors without masks and without socially distancing* | 03-08-2021 |
| More than 100 million COVID-19 vaccine doses have been administered in the U.S.* | 03-13-2021 |

*Table note:* The set of contextual events considered in this coordinated analysis are listed in the table. For simplicity, only the events marked with an asterisk above are listed in Figure 1; these were selected for relevance to the social interaction and loneliness topics of the manuscript as well as specific locations relevant for EAS, SRS, and WFDS datasets.

**COVID-19 Events Sources**

Centers for Disease Control and Prevention. (2023, March 15). COVID-19 Timeline. [Website]. URL:<https://www.cdc.gov/museum/timeline/covid19.html>

CNN. (2023, May 8). COVID-19 Pandemic: A Timeline of Fast Facts. CNN. URL:<https://www.cnn.com/2021/08/09/health/covid-19-pandemic-timeline-fast-facts/index.html>

Gorel, A. (2022, February 1). For the First Time in Two Years, Massachusetts Reports No New COVID-19 Cases. WBUR. URL:<https://www.wbur.org/news/2022/02/01/first-covid-two-years-massachusetts>

Infectious Disease Advisor. (2020, March 3). First Case of COVID-19 in NYC, First Death Reported in Washington State. Infectious Disease Advisor. URL:<https://www.infectiousdiseaseadvisor.com/home/topics/respiratory/first-case-of-covid-19-in-nyc-first-death-reported-in-washington-state/>

Michigan Department of Health & Human Services. (2020, March 10). Michigan Announces First Presumptive Positive Cases of COVID-19, Governor Whitmer Declares a State of Emergency. Michigan.gov. URL:<https://www.michigan.gov/coronavirus/news/2020/03/10/michigan-announces-first-presumptive-positive-cases-of-covid-19-governor-whitmer-declares-a-state-o>

**Supplementary Table 2.** Measures

| **Key Variable** | **Study** | | | | **Harmonization** |
| --- | --- | --- | --- | --- | --- |
|  | DCF | EAS | SRS | WFDS |  |
| *Loneliness* | Please indicate how often you have felt this way today by checking the appropriate box for each question: I felt lonely.  1=Rarely or none of the time  2=Some or a little of the time  3=Occasionally or a moderate amount of time  4=All of the time | Do you feel lonely?  Not at all (0) -- Extremely (100) | I felt lonely.  1 = Rarely or none of the time (< 1 day)  2 = some or a little of the time (1-2 days)  3 = occasionally or a moderate amount of time (3-4 days)  4 = Most or all of the time (5-7 days) | You felt lonely... Did you feel that way less than 1 day last week, 1-2 days, 3-4 days, or 5-7 days last week  1 = <1 day  2 = 1-2 days  3 = 3-4 days  4 = 5-7 days | Loneliness (at all measurement occasions): POMP Scores |
| *Social interaction* | Besides the people you live with, how many people have you interacted in person with in the past 24 hours?  [open response] | Have you had any social interactions? (An interaction is talking or spending time with someone in person, by phone/computer or by texting.)  0=No  1=Yes | How often do you see family and friends in person, not including people with whom you live?  1= Irregularly  2= Once a month or more often  3= once a week or more often  4= Every day | How often do you get together with your (mother/father/adult child) that is you visit (her/him), (she/he) visits you, or you go out somewhere together…?  1 = Never  2 = Less than once a month  3 = About once a month  4 = 2-3 times a month  5 = At least once a week  6 = Several times a week  7 = Every day | Social Interaction: POMP score |
| *Age* | Calculated from birth year | Age at wave | Calculated from birth year | Calculated from birth year | Centered at study mean |
| **Control Variables** |  |  |  |  |  |
| *Gender* | To which gender do you most identify?  0=Male  1=Female  2=Transgender Female  3=Transgender Male  4=Gender Variant/Non-Conforming  5=Not listed  6=Prefer not to answer | What is your current gender?  1=Male 2=Female 3=Transgender male 4=Transgender female 5=Other/Self-identified  6=Prefer not to answer | Are you male or female? | Provided by R’s mother at current or previous wave (depending on generational involvement) and confirmed with R during interview  [open response] | 0 = Male  1= Female |
| *Race / Ethnicity* | Please choose the category that best describes your racial background  0=White  1=Black or African American  2=Other  3=Asian  4=Native American | To which ethnic group do you belong?  1=Caucasian  2=African American  3=Hispanic, White  4=Hispanic, Black  5=Asian  6=Other | Please tell me which categories describe you. Are you American Indian or Alaska Native, Asian, Black, Hispanic or Latino, Middle Eastern, North African or Arab American, Native Hawaiian or Other Pacific Islander or White?  [multiple choice] | Which of the following do you consider yourself—please choose as many as you feel describe you  1=White  2=Black or African American  3=Asian  4=American Indian or Alaska Native  5=Native Hawaiian or Other Pacific Islander  6=Other [specify] | 0 = Non-White  1 = White |
| *Marital Status* | Partnership status (choose the one that best describes your partnership status)  1=Married  2=Separated  3=Divorced  4=Partnered (in a committed relationship but not married)  5=Windowed  6=Single | What is your current marital status?  1=Married  2=Separated  3=Widowed  4=Divorced  5=Never married | Are you currently married or living with a partner, widowed, divorced, separated, or have you never married?  [multiple choice] | What is your present marital status? Are you married, living with a partner, separated, divorced, widowed, or have you never been married?  [multiple choice] | 0 = All others  1 = Married or partnered |
| *Education* | How many years of education have you completed?  [open response] | How many years of education have you had?  [open response] | How many years of school have you completed?  [0-17] | What was the highest grade in school that (adult child) completed? (Reported by mother at previous or current wave)  1 = None  2 = Eighth grade or less  3 = 1-3 years of high school  4 = High school graduate  5 = Vocational/Non-college  6 = Post high school  7 = 1-3 years of college  8 = College degree  9 = Graduate work  10 = Other | z-scored |
| *Income* | Current household income level ($/year)  1=$10,000 or less  2=$10,000 to $25,000  3=$25,000 to $50,000  4=$50,000 to $100,000  5=$100,000 to $250,000  6=$250,000 or more | What is your current income?  1= Less than $15,000  2= Between $15,001 and $30,000  3= Greater than $30,000 4=Refused 5=Don’t know | Considering income from all sources-from jobs, remittances, pensions, interest, rents, and so forth-for you and all household members living with you, what would you say was your total household income last month?  1 = Less than $500  2 = $501-1000  3 = $1001-2000  4 = $2001-3000  5 = $3001-5000  6 = $5001-10,000  7 = More than $10,000 | What was (your/you and your (partner/spouse)’s) total income before taxes in 2019?  1 = Less than $30,000  2 = $30,000-$49,999  3 = $50,000-$74,999  4 = $75,000-$99,999  5 = $100,000-$149,999  6 = More than $150,000 | z-scored |
| *Employment Status* | Please choose the category that best describes your current employment situation  1=Working part-time  2=Working full-time  3=Unemployed  4=Temporarily laid off or furloughed  5=Stay-at-home parent  6=Homemaker  7=Student  8=Maternity or family leave  9=Permanently disabled  10=Retired  11=Other, please describe | What is your current work status?  1=Working for pay 2=Unemployed but looking for work 3=Unemployed and NOT looking for work 4=Homemaker 5=Retired | Are you currently working?  0= not working  1= working | Which of the following best describes your current employment status - unemployed and looking for work, disabled, taking care of your family and home, laid off, retired, or something else?  [multiple choice] | 0 = Not employed  1 = Employed |
| *Self-Rated Health* | How would you rate your overall health?  1=Poor  2=Fair  3=Average  4=Good  5=Excellent | Average score of Health1- Health14.  1 = Unable to do  2 = With much difficulty  3 = With some difficulty  4 = With a little difficulty 5 = Without any difficulty | How would you rate your health at the present time? Would you say it is excellent, very good, good, fair, or poor?  1=Poor  2=Fair  3=Average  4=Good  5=Excellent | Would you say that your physical health is excellent, very good, good, fair, or poor?  1=Poor  2=Fair  3=Average  4=Good  5=Excellent | z-scored |

*Table note*. For WFDS participants who entered the study in wave 3 (the Wave used for analyses) as the third generation of their family to participate in the WFDS, the measure of social interaction with their parents only reflects contact with the parent who participated in the WFDS (participants were not asked about contact with their non-participating parents, or with any offspring). For participants who are the second generation of their family to participate in the WFDS (i.e., whose mothers participated in Wave 1), they were asked about contact with any living parents and children. These respondents’ total in-person contact with their children was divided by the number of children in the family in order to create a measure of average contact with children, and for those with both living parents, contact with parents was also averaged. Regardless of earlier generations’ participation, any participant without any living parents or adult children were coded 1 (i.e.,”never”). Finally, contact with their parents (the first generation of participants) and their children (the third generation of participants) was averaged.

**Supplemental Table 3. Unadjusted Results from Four Studies - Loneliness predicted by Social Interactions, Age, and their Interaction**

| *Fixed Effects* | Multi-Level Models | | | | | | OLS Regression Models | | | | | |
| --- | --- | --- | --- | --- | --- | --- | --- | --- | --- | --- | --- | --- |
|  | DCF | | | EAS | | | SRS | | | WFDS | | |
|  | Est | SE | *p* | Est | SE | *p* | Est | SE | *p* | Est | SE | *p* |
| Intercept | 1.87*** | 0.17 | < .0001 | 1.75 | 0.1403 | <.0001 | 2.69** | 0.40 | <.0001 | 1.899*** | 0.186 | <.0001 |
| BP Social Interactions | -0.12 | 0.21 | 0.55 | -2.19 | 0.7117 | 0.0024 | -0.07 | 0.08 | 0.36 | -0.029 | 0.029 | 0.325 |
| WP Social Interactions | -0.04 | 0.03 | 0.17 | -0.14 | 0.02454 | <.0001 | - | - | - | - | - | - |
| Age | -0.03** | 0.01 | 0.01 | -0.01 | 0.02726 | 0.6245 | 0.00 | 0.06 | 0.95 | -0.003 | 0.011 | 0.805 |
| Age X BP Social Interactions | -0.003 | 0.01 | 0.83 | 0.12 | 0.1323 | 0.3641 | 0.01 | 0.01 | 0.40 | -0.003* | 0.002 | 0.041 |
| Age X WP Social Interactions | 0.002 | 0.00 | 0.23 | 0.01 | 0.004925 | 0.0037 | - | - | - | - | - | - |
| *Random Effects* |  |  |  |  |  |  |  |  |  |  |  |  |
| Intercept (person) | 5.78*** | 0.59 | < .0001 | 3.30 | 0.52 | <.0001 | - | - | - | - | - | - |
| Intercept (day) | - | - | - | 0.36 | 0.02 | <.0001 | - | - | - | - | - | - |
| Cov(Int,WP Social Interaction Slope) | *-^a* | - | - | -3.45 | 1.16 | 0.0031 | - | - | - | - | - | - |
| WP Social Interaction Slope | *-^a* | - | - | 7.94 | 8.01 | 0.1607 | - | - | - | - | - | - |
| Residual | 2.63*** | 0.07 | < .0001 | 0.94 | 0.01 | <.0001 | - | - | - | - | - | - |
| N(Persons) | 217 |  |  | 195 |  |  | 141 |  |  | 801 |  |  |
| N(Observations) | 3229 |  |  | 14738 |  |  | - |  |  | - |  |  |

*Table note.* BP=Between person; WP=Within person. Social interactions were POMP scored. Age was centered at each study’s mean. Results for DCF and EAS are drawn from multilevel models of intensive longitudinal data, thus these include separate predictors for the between person and within person effects of social interactions. Within person effects were person-mean centered; between person effects were grand mean centered. These models include random effects, except where indicated with an *^a*. The model for DCF which included a random slope did not converge, thus the reported model includes only a random intercept. Results for SRS and WFDS are drawn from OLS regression of cross-sectional surveys, thus these include the between person effect of social interactions, and no random effects.

**Supplemental** **Table 4. EAS Results Including Self-Rated** **Health**

|  | *Est* | SE | *p* |
| --- | --- | --- | --- |
| *Fixed Effects* |  |  |  |
| Intercept | 0.63 | 0.85 | 0.4563 |
| Social Interactions: BP | -1.79* | 0.77 | 0.0214 |
| Social Interactions: WP | -0.18** | 0.05 | 0.0001 |
| Age | 0.02 | 0.04 | 0.5679 |
| Age X Social Interaction BP | 0.05 | 0.12 | 0.6795 |
| Age X Social Interaction WP | 0.02 | 0.01 | 0.0509 |
| Sex | 0.92 | 0.42 | 0.0295 |
| Race | -0.03 | 0.36 | 0.9393 |
| Marital Status | -0.09 | 0.45 | 0.8403 |
| Education | -0.36 | 0.19 | 0.0652 |
| Income | 0.29 | 0.20 | 0.1427 |
| Employment | 0.93 | 0.78 | 0.2346 |
| Self-Rated Health | -0.15 | 0.19 | 0.4124 |
| *Random Effects* |  |  |  |
| Intercept (person) | 3.68 | 0.48 | <.0001 |
| Intercept (day) | 0.30 | 0.02 | <.0001 |
| Cov(Int,WP Social Interaction Slope) | -0.18 | 0.10 | 0.0582 |
| WP Social Interaction Slope | 0.13 | 0.03 | <.0001 |
| Residual | 0.94 | 0.02 | <.0001 |
| Persons | 129 |  |  |
| Observations | 10163 |  |  |

*Table note.* BP: Between person; WP: Within person. In Table 3 in the main text, we excluded self-rated health from the model in order to conduct analyses on the full sample who provided data during the period of study (N=194). The present table presents results of the model with self-rated health for the N=129 EAS participants who had available data for self-rated health. Social interactions were POMP scored. Age was centered at the study’s mean. Results are drawn from multilevel models of intensive longitudinal data, thus these include separate predictors for the between person and within person effects of social interactions. Within person effects were person-mean centered; between person effects were grand mean centered. These models include random effects.

**Section 2: Method Details--Individual Study Analysis**

All models were adjusted for sex, race, marital status, education, income, employment status, and self-rated health. We applied multilevel models (MLMs) to the intensive repeated-measures datasets (i.e., DCF, EAS) with nested data. The two-level equations used in DCF and the three-level equations for EAS momentary data are shown below. First, we estimated unconditional MLMs to partition the variance in social interaction and loneliness using the following equations:

Level 1 (within-person): social interaction = β0 + r

Level 2 (between-person): β0 = γ00 + u0

Level 1 (within-person): loneliness = β0 + r

Level 2 (between-person): β0 = γ00 + u0

We then estimated conditional multilevel models to test age differences in the between-person and within-person relationships between social interaction and loneliness:

Level 1 (within-person): loneliness = β0 + β1(social interaction) + r

Level 2 (between-person):

β0 = γ00 + γ01(age) + γ02(social interaction) + γ03(social interaction*age) + u0

β1 = γ10 + γ11(age) + u1

For the cross-sectional studies (i.e., SRS, WFDS), we estimated ordinary least squares (OLS) regression models where:

Loneliness = intercept + b1(social interaction) + b2(age) + b3(social interaction*age)
